# Unique synergistic formulation of curcumin, epicatechin gallate and resveratrol, tricurin, suppresses HPV E6, eliminates HPV+ cancer cells, and inhibits tumor progression

**DOI:** 10.18632/oncotarget.16648

**Published:** 2017-03-29

**Authors:** Sumit Mukherjee, Priya Ranjan Debata, Rahman Hussaini, Kaushiki Chatterjee, Juliet N.E. Baidoo, Samay Sampat, Anita Szerszen, Joseph P. Navarra, Jimmie Fata, Elena Severinova, Probal Banerjee, Mario R. Castellanos

**Affiliations:** ^1^ Department of Chemistry, The College of Staten Island (CUNY), New York, NY, USA; ^2^ CUNY Doctoral Program in Biology, CUNY Graduate Center, New York, NY, USA; ^3^ CUNY Doctoral Program In Biochemistry, CUNY Graduate Center, New York, NY, USA; ^4^ Department of Biology, The College of Staten Island (CUNY), New York, NY, USA; ^5^ Center of Developmental Neuroscience, The College of Staten Island (CUNY), New York, NY, USA; ^6^ Division of Research, Department of Medicine, Staten Island University Hospital (Northwell Health), New York, NY, USA; ^7^ College of Pharmacy and Health Sciences, St. John’s University, New York, NY, USA; ^8^ Current Address: Department of Zoology North Orissa University Baripada, Mayurbhanj, Odisha, India; ^9^ Current Address: Cell Biology and Molecular Medicine, Rutgers University, Newark, NJ, USA

**Keywords:** curcumin, cervical cancer, human papillomavirus, synergism, combination index

## Abstract

Curcumin (from curry) (C) is highly potent against cervical cancer cells (CCC), but poor bioavailability has limited its clinical use. Similar natural polyphenols resveratrol (from grapes) (R), and epicatechin gallate (from green tea) (E) also display activity against CCC. By treating CCC (HeLa) with C, E, or R, or combinations of these compounds, we computed combination indices and observed a strong synergism among C, E, and R at the unique molar ratio 4:1:12.5. This combination, named as TriCurin, rapidly down regulated HPV18 E6 and NF-kB expression while concomitantly inducing the tumor suppressor protein p53 in HeLa cells. In the mouse c-Ha-ras and HPV16 E6, E7-expressing TC-1 CCC, both C and TriCurin elicited suppression of E6, induction of both p53 and acetyl-p53 (activated p53), and activation of caspase-3, but the TriCurin-evoked changes were several-fold greater than that produced by curcumin (4.7-fold for E6 inhibition, and 2-fold, 6-fold, and 1.7-fold for the induction of p53, acetyl-p53, and active caspase-3, respectively). Consequently, TriCurin was more potent in killing TC-1 and HeLa cells. Intralesional TriCurin treatment of tumors generated in mice by subcutaneously implanting the TC-1 CCC caused an 80–90% decrease in tumor growth. The ability of C to eliminate HeLa cells was significantly stabilized when delivered as TriCurin than when delivered alone. Topical application of TriCurin dispersed in a cream base afforded efficient transfer of C across the skin. Subcutaneous TriCurin injection yielded no adverse effect in tumor-naïve healthy mice. Thus, TriCurin is a safe and promising therapeutic agent against HPV-associated disease.

## INTRODUCTION

The human papillomavirus (HPV) is the prime risk factor for cervical cancer that claims numerous lives worldwide and poses a major threat to females especially in the developing countries [1]. Majority of sexually active women acquire HPV by the age of 50, which makes cervical HPV infection the most common sexually transmitted disease in the U.S. [2]. The incidence of cervical cancer is rather low in the developed countries because of extensive screening programs, but the prohibitive cost of these programs make them inappropriate for the developing world [3]. Although the availability of two vaccines, Cervarix^®^ or Gardasil^®^ [4], offer prophylactic measures against the most common oncogenic HPV types 16/18-associated cervical lesions, effective therapeutic measures for post-infection lesions are not currently available. Furthermore, such vaccination programs are not suitable for developing countries with limited resources.

During the past decade, our group has been developing diverse strategies of potentiating the culinary component, curcumin (C), against cancer cells in culture and also in mouse models of melanoma, glioblastoma, and HPV+ cervical cancer [5–9]. However, due to its hydrophobicity and rapid breakdown *in vivo*, curcumin *per se* has not been an effective therapeutic agent [10, 11]. Using our expertise in targeting curcumin we initially addressed the global menace of HPV infection and cervical cancer by developing and testing a curcumin-based cervical cream and demonstrated its efficacy in eliminating cervical cancer cells [5]. In the current study, we have further potentiated the antitumor efficacy of curcumin through a simple strategy, which involves mixing of curcumin with two other polyphenols, epicatechin gallate (E) and resveratrol (R), at a unique synergistic molar ratio. As an ingredient of green tea, epicatechin gallate displays toxicity toward a wide range of cancer cells [12], and, unlike curcumin, it is soluble in water. An important component of grapes, resveratrol is known for its anti-oxidant, anti-inflammatory, and anti-cancer properties [13]. Earlier studies have also shown that resveratrol functions in synergism with curcumin against cancer cells [14]. Our unique mixture of C, E, and R (named as TriCurin) shows sharply increased antitumor effects both *in vitro* as well as *in vivo* and can be applied in a cream base as a safe anticancer agent that readily permeates through the skin. Thus, we report here the design, mechanism of action, and application of a potentiated form of curcumin, TriCurin, as a promising therapeutic agent against HPV-associated neoplasia.

## RESULTS

### Combination index measurement to obtain a synergistic ratio of C, E, and R

**Figure 1:**
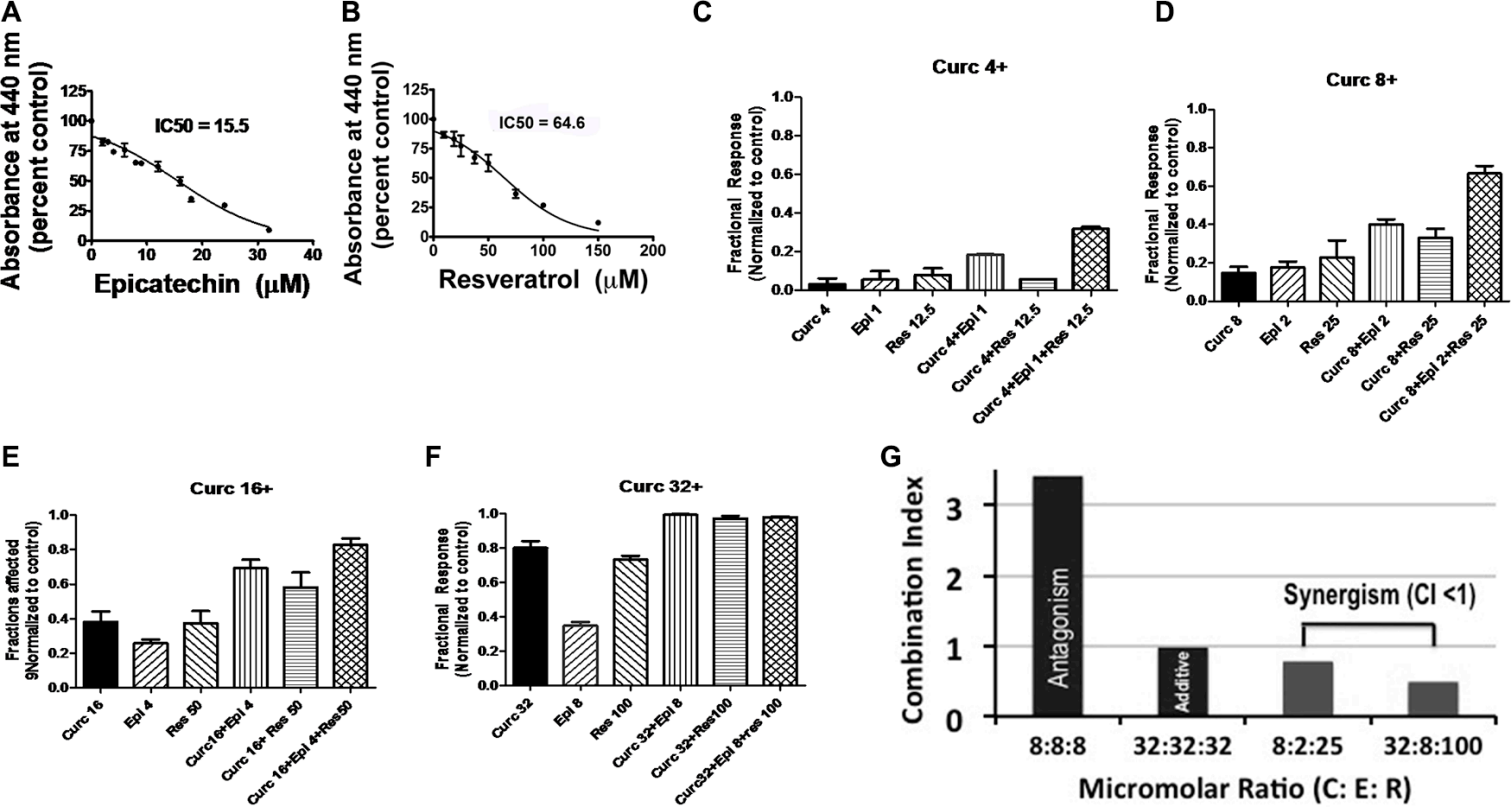
Fraction of cells affected by C, E, R, CE, CR, and CER: measurement of combination index to identify a synergistic formulation (**A**) and (**B**) Concentration-dependent elimination of HeLa cells by epicatechin gallate (E) and resveratrol (R), respectively. (**C**–**F**) Fractions of HeLa cells killed by CER at 4 μM+, 8 μM+, 16 μM+, 32 μM+, and the corresponding concentrations of C, E, R, CE, and CR. (**G**) Combination index (CI) >> 1 for equimolar proportion of C:E:R: 8 μM:8 μM:8 μM (the components antagonize one another). At CI = 1 for C:E:R: 32 μM:32 μM:32 μM, the components had an additive effect. At CI < 1 for C:E:R: 8 μM:2 μM:25 μM and C:E:R: 32 μM:8 μM:100 μM, the components are synergistic.

The IC50 for C (17 µM) for HeLa cells was obtained earlier using WST-1 assays [5]. In the current study, we similarly obtained 15.5 μM and 64.6 μM as IC50 values for E and R (for HeLa cells), respectively (Figure 1A and 1B). Next, we conducted several pilot experiments to determine the vulnerability of HeLa cells to those concentrations of C, E, and R that were at or below their individual IC50 values. Our initial experiments using a mixture of C, E, and R at their IC50 concentrations revealed toxicity even toward normal human fibroblasts. Then, by using sub-IC50 concentrations of E while holding the concentration of R at 100 µM a ratio of C:E:R: 32 µM: 8 µM: 100 µM (or C:E:R in the molar ratio of 4:1:12.5) was obtained that showed selective toxicity toward cancer cells. At doses C:E:R: 4:1:12.5, C:E:R: 8:2:25, and C:E:R: 16:4:50, the fractions of HeLa cells affected by the combination was greater than that observed with each of the individual components C, E, or R or the double combinations CE or CR (Figure 1C, 1D, and 1E). This ratio was named as TriCurin and the increasing doses of C:E:R: 4:1:12.5, C:E:R: 8:2:25, C:E:R: 16:4:50, and C:E:R: 32:8:100 were dubbed as 4 µM+, 8 µM+, 16 µM+, and 32 µM+ respectively. We next analyzed our data in the fraction of HeLa cells affected (killed) through Combination Index (CI) determination using the COMPUSYN software package from ComboSyn, Inc. (www.combosyn.com) and a strategy reported by Chou [15]. A synergism among the components of TriCurin at 8 µM+ and 32 µM+ was revealed when the CI at each of these doses was found to be less than 1 (Figure 1G). In sharp contrast, equal proportions of C, E, and R were either antagonistic (CI >> 1) or additive (CI = 1) (Figure 1G).

### TriCurin is more potent than curcumin in killing both TC-1 and HeLa cells

In WST-1 assays, TriCurin displayed a 2.7-fold lower IC50 than C alone for the c-Ha-ras and HPV16 E6, E7-expressing mouse TC-1 cervical cancer cells [16] and a 4-fold lower IC50 than C alone for HeLa cells (Table 1).

**Table 1:** Tricurin is more potent in eliminating both TC-1 and HeLa cells

Cell Lines	Curcumin	Tricurin
	IC50 (µM)	IC50 (µM)
TC-1	35	13
HeLa	17	4

### TriCurin treatment boosts p53 expression, suppresses NF-kB, and suppresses E6 expression in HeLa cells

**Figure 2:**
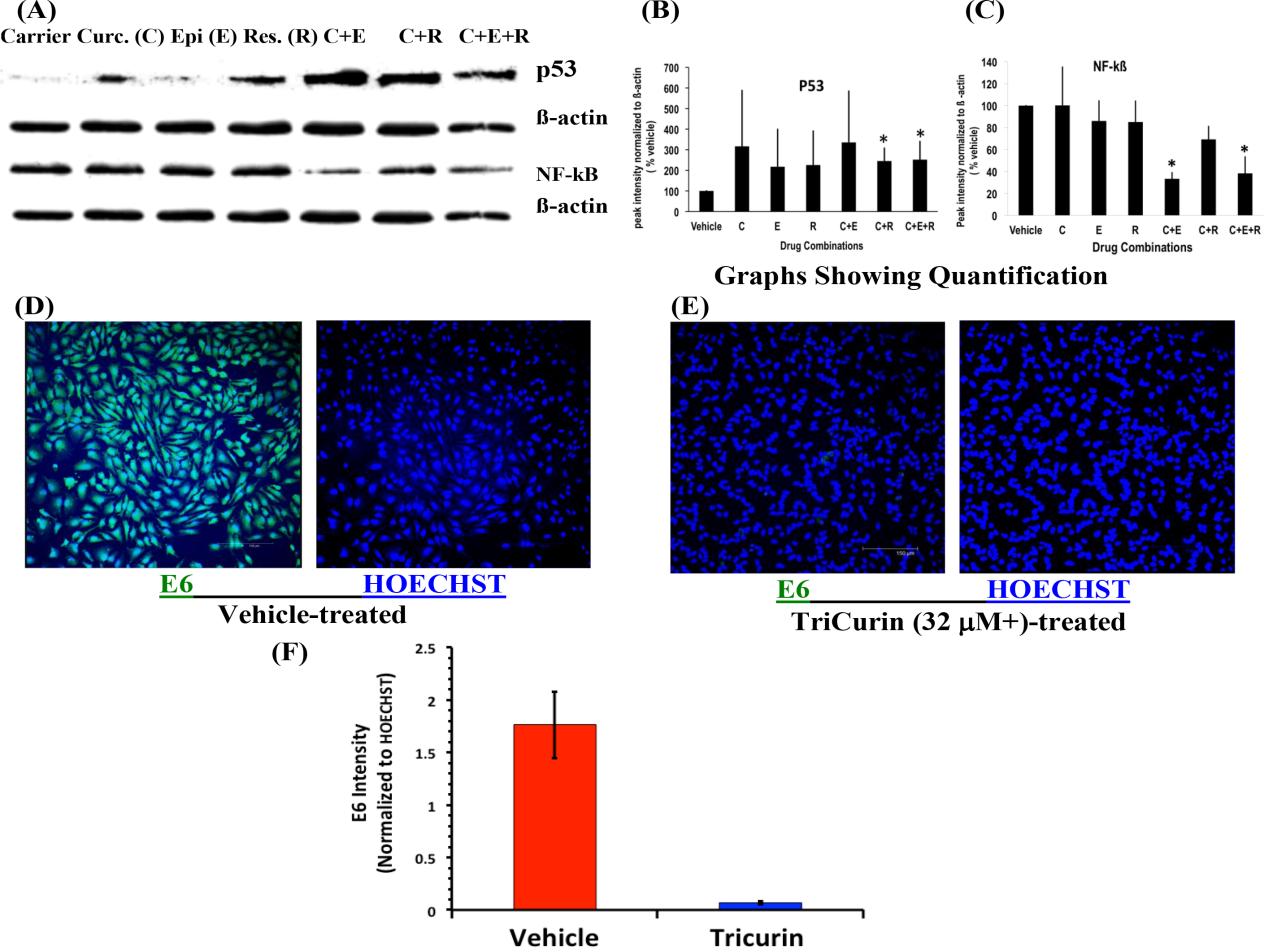
TriCurin causes restoration of p53 and suppression of NF-kB and E6 in HeLa cells HeLa cells in culture were treated with drugs for 8 h for Western blotting and for 6 h for immunocytochemistry. (**A**) and (**B**) Cells treated with the corresponding CR and CER (32 μM+ TriCurin) show a significant increase in p53 expression. (A) and (**C**) Cells treated with CE and CER (32 μM+ TriCurin) display a significant decrease in NF-kB expression in (*p < 0.05). (**D**–**F**) E6 expression is significantly suppressed following 32 μM+ TriCurin treatment (*p* = 0.0008) (Data expressed as mean ± S.E.M).

We have shown earlier that 50-µM curcumin treatment causes a dramatic induction of the cell cycle inhibitor p53 and suppression of the pro-tumor protein NF-kB in the HeLa cells [5]. In these experiments, we observed only a partial induction of p53 at 32 µM C alone. However, delivering 32-µM C along with 8-µM E and 100-µM R (i.e. TriCurin) caused a more dramatic induction of p53 within 8 hours (Figure 2). In this case even the combinations CE and CR caused an induction of p53, but from three experiments, the induction in p53 was most significant with TriCurin and CR (Figure 2B). Concomitantly, a sharp decrease in NF-kB expression was observed in the HeLa cells following treatment with TriCurin or CE (Figure 2C). Immunocytochemical analysis of TriCurin-treated HeLa cells revealed a dramatic inhibition of HPV E6 expression (Figure 2D–2F).

Although we initially used the human cervical cancer cell line HeLa to invent the potentiated formulation of curcumin (TriCurin), our major objective was to test the anticancer efficacy of TriCurin in a mouse model implanted with the mouse c-Ha-ras and HPV16 E6, E7-expressing TC-1 cells [16]. Therefore, it was imperative for us to delineate the possible mechanism of TriCurin-evoked elimination of TC-1 cells. In normal cells, the histone acetyltransferase (HAT) p300 and its co-activator CBP bind to the cell cycle inhibitor P53 to cause its activation through p300-mediated acetylation [17]. In HPV+ cancer cells, the oncoprotein E6 disrupts this P53-p300-CBP interaction by binding to both CBP and p300 and rendering them less effective to cause p53 activation [18]. Thus, suppression of E6 expression in the cancer cells would be expected to trigger activation of p53 through p300-mediated acetylation of p53, and p53-dependent expression of Bax, which triggers apoptosis [19]. We treated cultured TC-1 cells with vehicle, 32-μM+ TriCurin, or 32-μM curcumin and performed a mechanistic analysis by immunostaining for E6, active caspase-3, p53, and acetyl-p53 (Supplementary Figure S1).

### TriCurin is significantly more potent than curcumin in causing suppression of E6 and activation of caspase-3 in TC-1 cells

**Figure 3:**
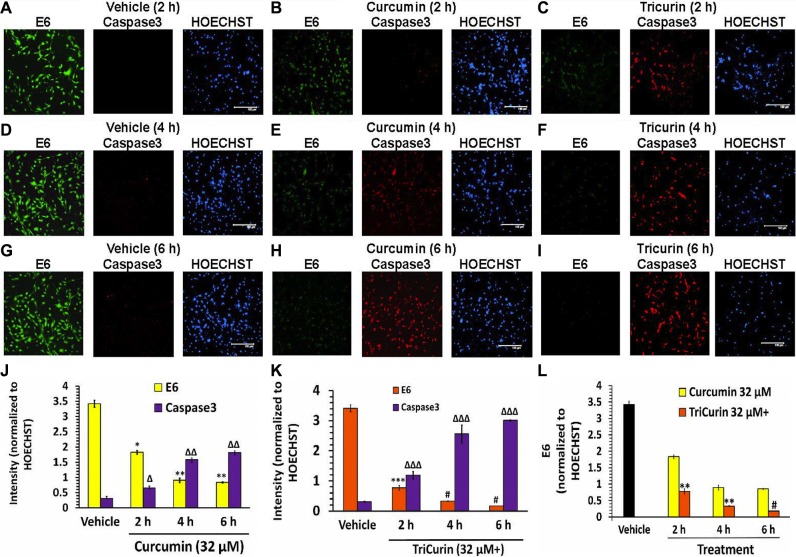
TriCurin is more potent than curcumin in causing suppression of E6 and activation caspase-3 in TC-1 cells TC-1 cells in triplicate wells were treated with vehicle, or 32-μM curcumin, or 32-μM+ TriCurin for 2, 4, and 6 hours, fixed, and then subjected to immunocytochemistry. (**A**, **D**, **G**) High E6 expression (green) and negligible active caspase-3 (red) observed after 2, 4, and 6 h of vehicle treatment. (**B**, **E**, **H**) Curcumin elicits a pronounced decrease in E6 and a dramatic increase in active caspase-3. (**C**, **F**, **I**) TriCurin yields a more dramatic suppression of E6 and increase in caspase-3 than curcumin. Scale bar: 150 μm. (**J**–**L**) Quantification of staining intensities from triplicate samples (two images per sample) and normalization to the corresponding HOECHST staining intensities shows a time-dependent decrease in E6 and a progressive increase in active caspase-3 following curcumin or TriCurin treatment. Both changes appear significantly more pronounced with TriCurin. *P*-values: *, **, or ***, or #: E6 levels after drug treatment compared to vehicle-treated control (J and K) or when comparing between curcumin and TriCurin (L). Δ, Δ Δ, or Δ Δ Δ: active caspase-3 levels after drug treatment with respect to vehicle-treated control (J and K). *Δ: *p* <0.04 **Δ Δ: *p* < 0.02 ***Δ Δ Δ: *p* < 0.03; #*p* < 0.01. Data expressed as mean ± S.E.M.

As expected based on the mechanisms discussed above, compared to the vehicle-treated TC-1 cells, TriCurin (32 μM+) treatment for 6 h caused a 19-fold inhibition of E6 and a 9.7-fold activation of the mediator of apoptosis caspase-3 (Figure 3C, 3F, 3I, 3J–3L). Although 32-μM curcumin alone also caused E6 suppression and caspase-3 activation, 32-μM+ TriCurin elicited 4.7-times greater suppression of E6 (Figure 3L) and 1.7-fold higher activation of caspase-3 (Figure 3B, 3E, 3H–3K) than 32-μM curcumin alone after 6-h of treatment.

### TriCurin is significantly more potent than curcumin in activating P53 in TC-1 cells

**Figure 4:**
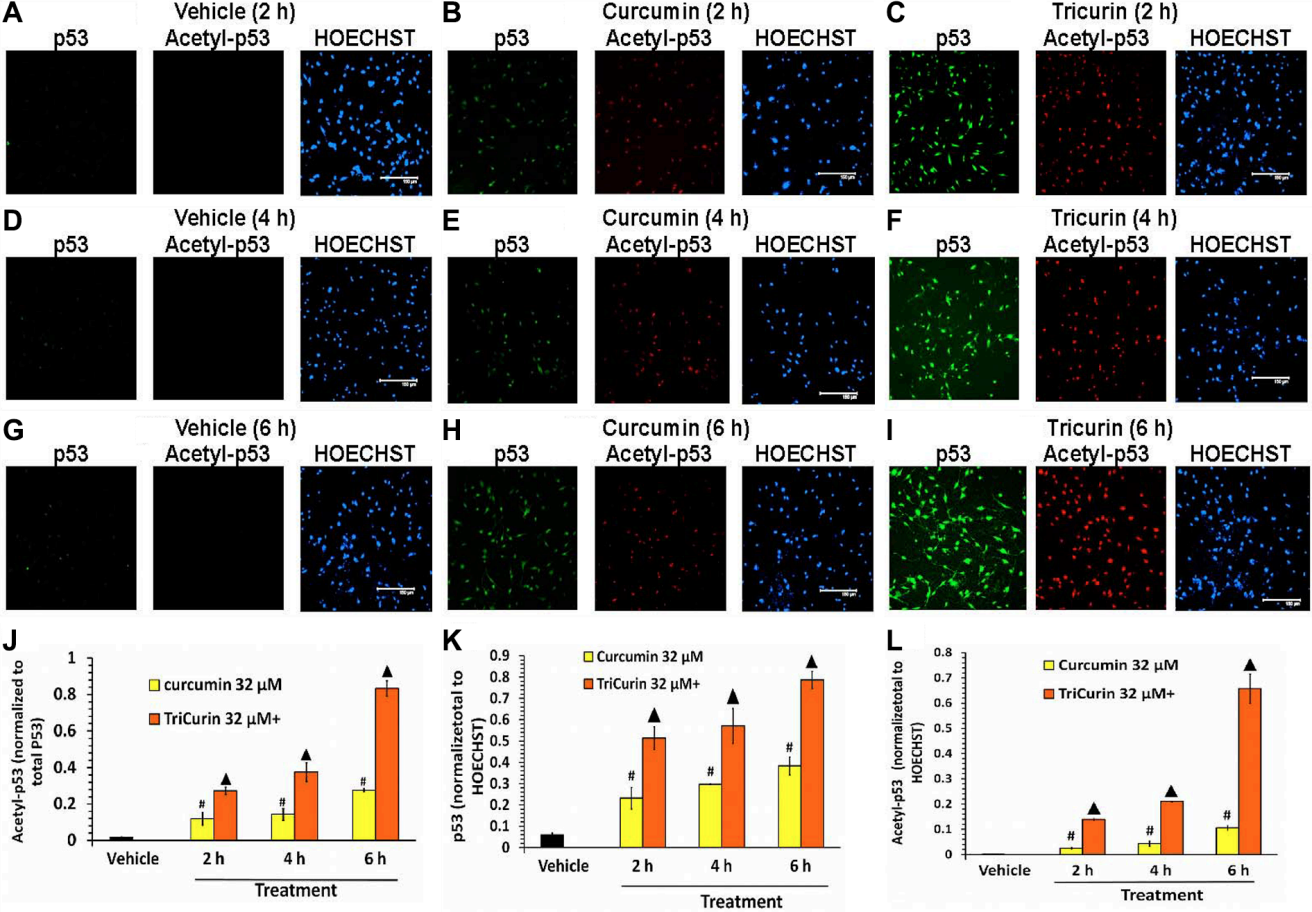
TriCurin is more potent than curcumin in regulating p53 in TC-1 cells After vehicle or drug treatment of TC-1 cells (as described in Figure 3), the cells were probed for p53 (green) and acetyl-^379^K-p53 (red). (**A**, **D**, **G**) Vehicle-treated cells show virtually no p53 or acetyl-p53. (**B**, **E**, **H**) Curcumin treatment causes an increase in both p53 as well as acetyl-p53 over time. (**C**, **F**, **I**) TriCurin elicits a more dramatic increase in both p53 as well as acetyl-p53 compared to curcumin. Scale bar: 150 μm. (**J**) Normalization of acetyl-p53 to total p53 reveals a progressive increase in acetylation of p53 (activation) after curcumin or TriCurin treatment (compared to vehicle-treated control), but TriCurin elicits a three-fold higher activation than curcumin after 6 h. (**K**) A pronounced increase in p53 expression (induction) is observed compared to the vehicle-treated control in both curcumin as well TriCurin-treated cells, but a two-fold higher induction is observed with TriCurin than with curcumin after 6 h of treatment. (**L**) The combination of activation and induction of p53 yields an overall induction in active p53 after either curcumin or TriCurin treatment. TriCurin yields a six-fold higher induction in active p53 than curcumin after 6 h (*p* < 0.005). *P*-values: (compared to vehicle-treated control): #*p* < 0.01; ▲: *p* < 0.005. Data expressed as mean ± S.E.M.

Concomitant with E6 suppression, 32-μM+ TriCurin treatment for 6 h caused an induction as well as activation of P53 (Figure 4C, 3F, 3I, 3J–3L). Thus, compared to vehicle-treatment, TriCurin treatment elicited a 13-fold induction of P53 (P53 staining normalized to HOECHST) (Figure 4K), and a 49-fold activation of P53 (Acetyl P53 staining normalized to P53 staining) (Figure 4J), thereby resulting in a 637-fold increase in the overall level of activated P53 (acetyl-P53 normalized to HOECHST) (Figure 4L). Although, compared to vehicle treatment, 32-μM curcumin treatment of TC-1 cells also caused an induction and activation of P53 (Figure 4B, 4E, 4H, 4J–4L), at 6 h, TriCurin treatment yielded a two-fold higher induction of p53 (Figure 4K), and a three-fold higher activation of p53 (Figure 4J), thereby eliciting a six-fold higher level of activated p53 than yielded by 32-μM curcumin alone (Figure 4L).

### Intralesional administration of TriCurin causes a dramatic inhibition of TC-1 tumors

**Figure 5:**
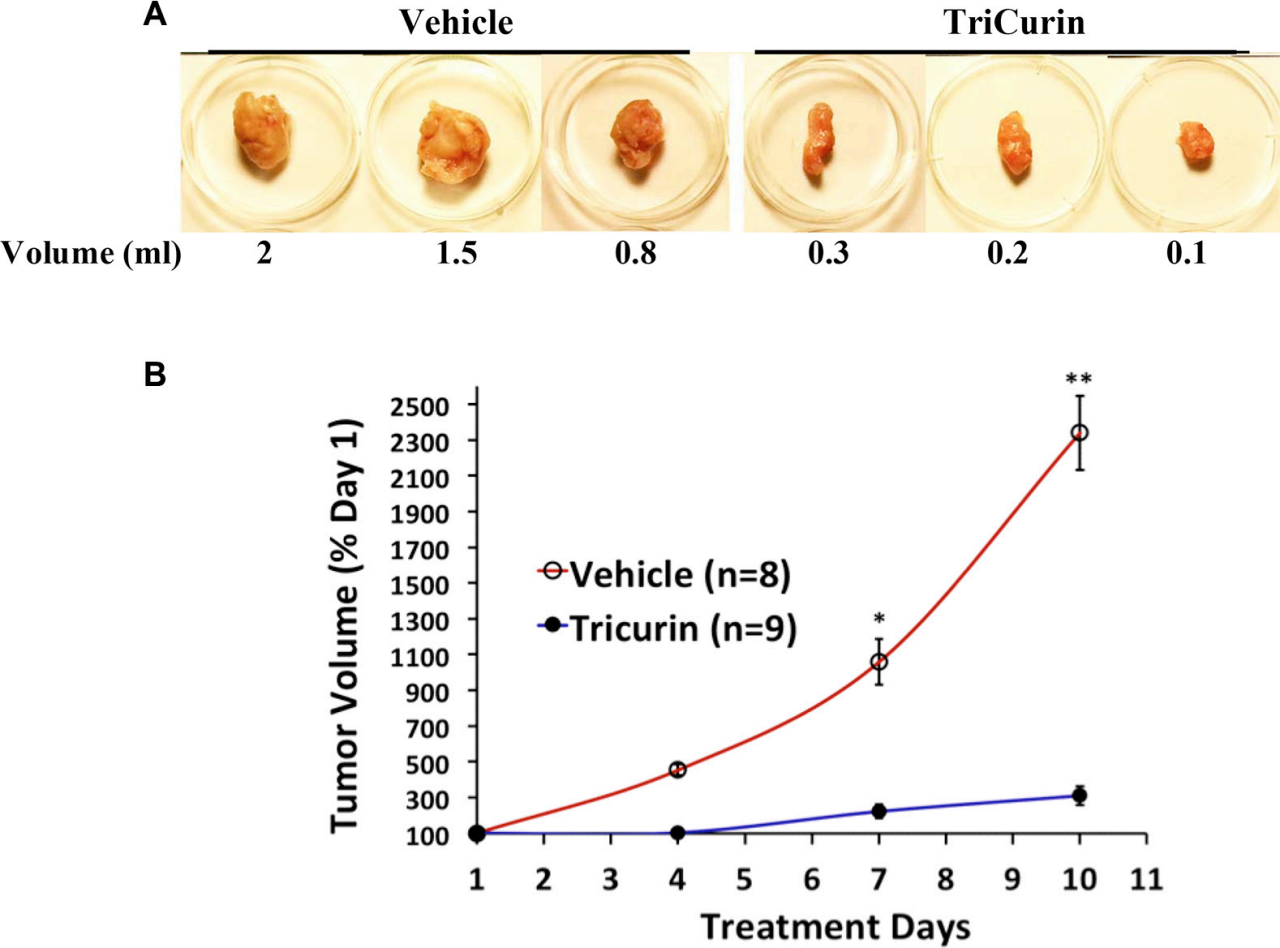
Intralesional TriCurin treatment causes a dramatic inhibition of TC-1 tumor growth in mice (**A**) Volumes of extricated tumors from vehicle-treated and 64 μM+ TriCurin-treated mice (as measured by the displacement of water on the day of sacrifice) (day 10). The largest, intermediate, and smallest tumors in each group are shown here. (**B**) Estimated volumes from external measurement of tumors from vehicle-treated (*n* = 8) and TriCurin-treated (*n* = 9) mice. *p = 0.0003. **p = 0.00001 (Data compared between vehicle-treated and TriCurin-treated on each day by two-tailed *t*-test with unequal variance and expressed as mean ± S.E.M).

Anti-tumor potency of TriCurin was demonstrated *in vivo* using a mouse model implanted (subcutaneously) at the nape of the neck with the mouse cell line TC-1. Based on the variation among volumes calculated from external dimensions of tumors and from water displacement of extricated tumors, intralesional administration of TriCurin into growing tumors was observed to cause an 80–90% decrease in tumor growth (Figure 5).

### The cytotoxic activity of curcumin toward HeLa cells is stabilized when present as a mixture in TriCurin

**Figure 6:**
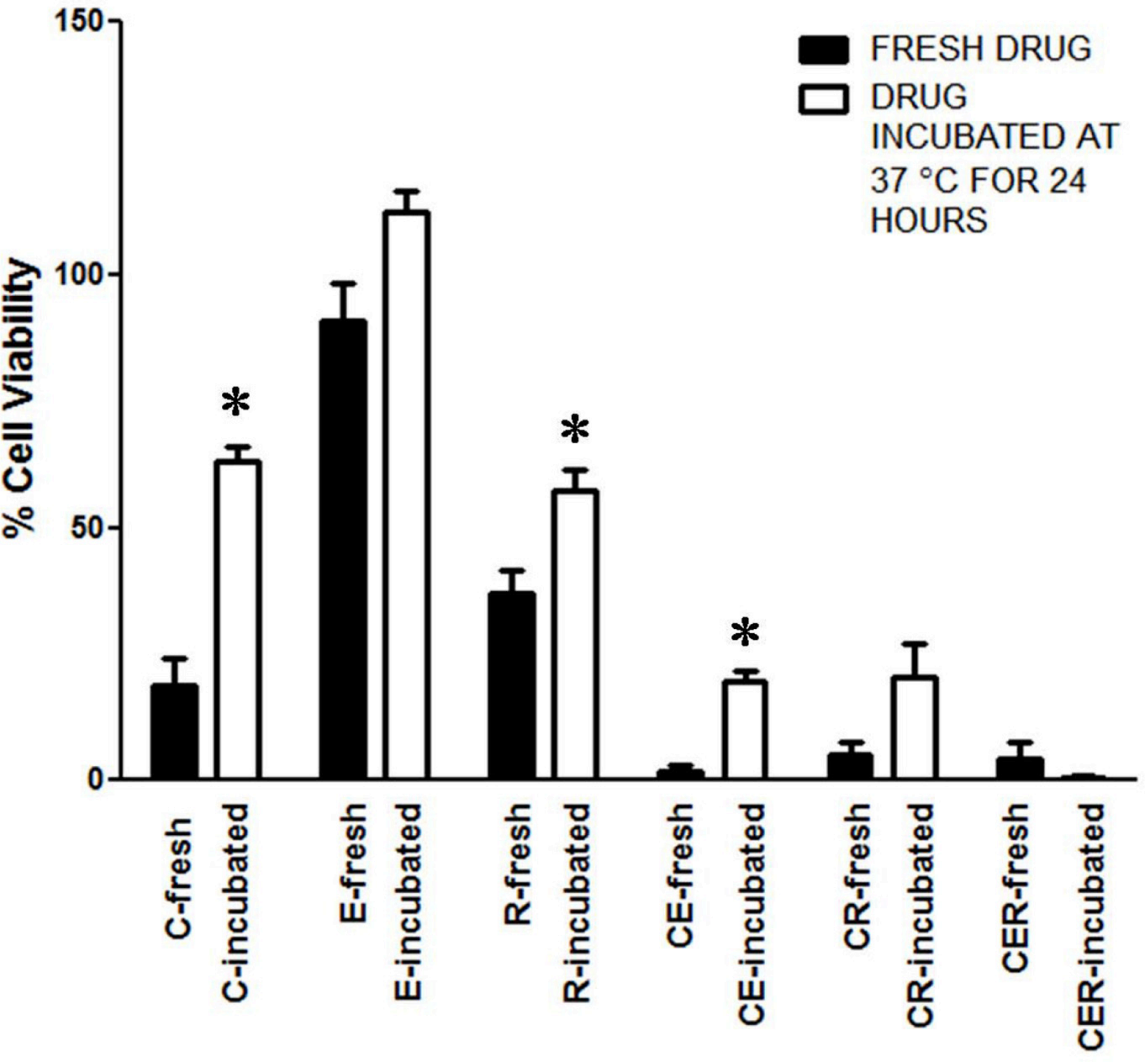
Epicatechin gallate and resveratrol stabilize curcumin’s ability to eliminate HeLa cells Freshly prepared media containing C (32 μM), E (8 μM), R (100 μM), CE, CR, CER, and an identical set of media incubated at 37°C for 24 h were used to treat HeLa cells for 48 h followed by WST-1 assay. Data shown are representative of three independent experiments, each of which was performed using triplicate samples and the results were expressed as mean ± SEM (*p ≤ 0.05 with respect to the effect of the freshly-prepared drug). The differences between the effects of freshly-prepared and incubated CER and also between incubated C and incubated CER were insignificant (two-tailed *t*-test with unequal variance).

In order to test if the antitumor activity of C was stabilized in the presence of E and R in TriCurin, we incubated C alone or as TriCurin in serum-free medium followed by treatment of HeLa cells with the medium. C, incubated as TriCurin, but not as CE or CR or C alone retained most of its potency to kill HeLa cells after 24 hours (Figure 6).

Additionally, we used the fluorescence from C at 540 nm (emission) to image cells. We observed that C from TriCurin was taken up by HeLa cells more rapidly than from C alone (Supplementary Figure S2- Supplementary Materials). Furthermore, following incubation of C or TriCurin in the culture medium overnight at 37°C, C from TriCurin was taken up more efficiently than from C alone (Supplementary Figure S1). These *in vitro* data support the use of TriCurin as an efficient strategy of delivering curcumin.

### TriCurin administration into tumor-naïve mice causes no toxic effect

**Figure 7:**
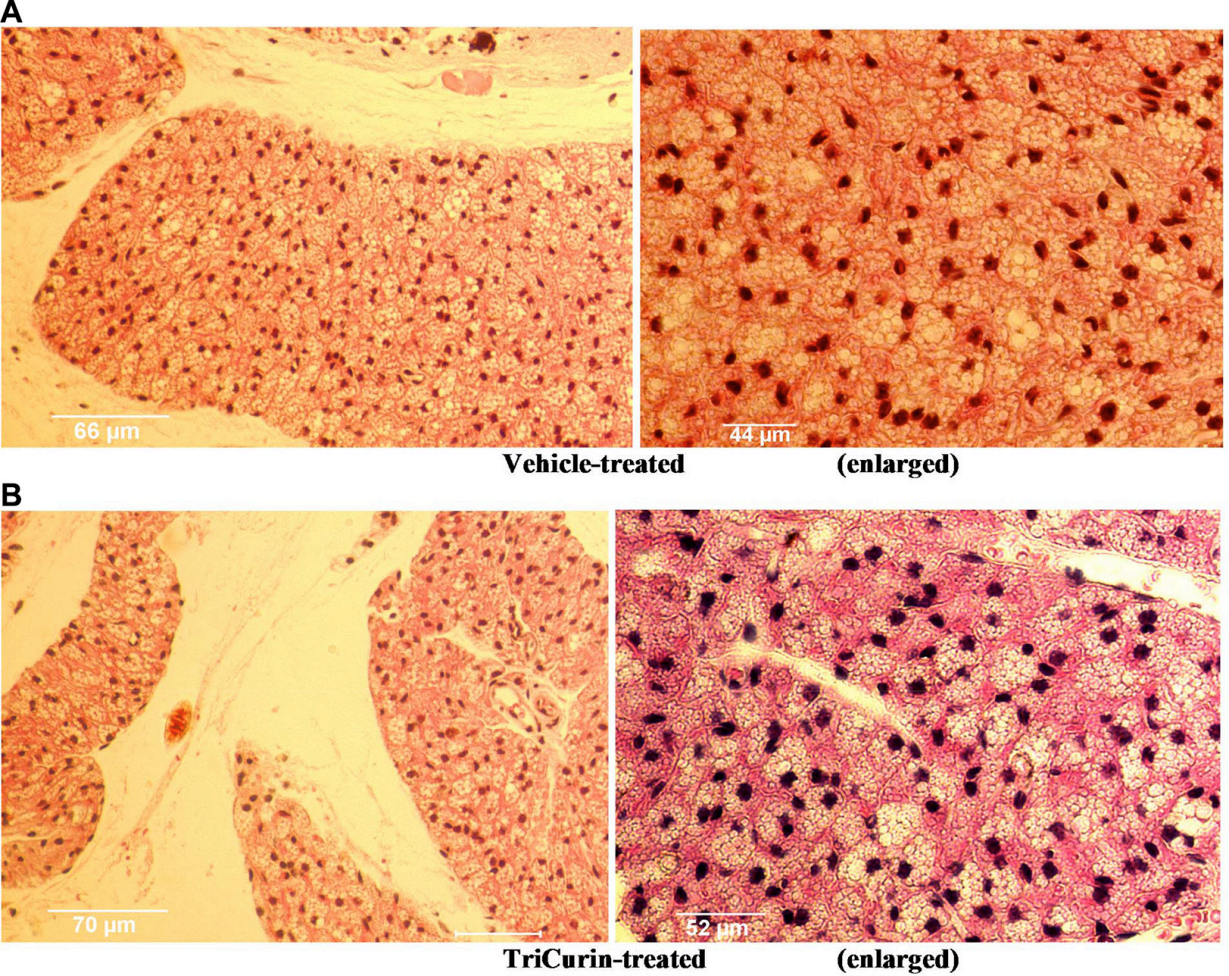
TriCurin is non-toxic to tumor-naïve mouse tissue H&E staining of tissue sections from the neck region of tumor-naïve mice. (**A**) Vehicle treated mouse. (**B**) Mouse injected subcutaneously five times, every 72 hours, with 1.28 mM+ TriCurin (at the nape of the neck). Enlarged views shown on the right.

To test if TriCurin administration had any toxic effect on normal tissue, tumor-naïve mice were injected with vehicle or TriCurin (10 µl of 1.28 µM+ TriCurin) subcutaneously at the nape of the neck, every 72 hours for two weeks. Dissection of the injection area followed by H&E staining and pathologic examination revealed no sign of heightened cell death following TriCurin treatment as compared to the vehicle-injected tissue (Figure 7).

### Topical application of a cream formulation of TriCurin allows permeation of curcumin through the skin layer within 20 hours

**Figure 8:**
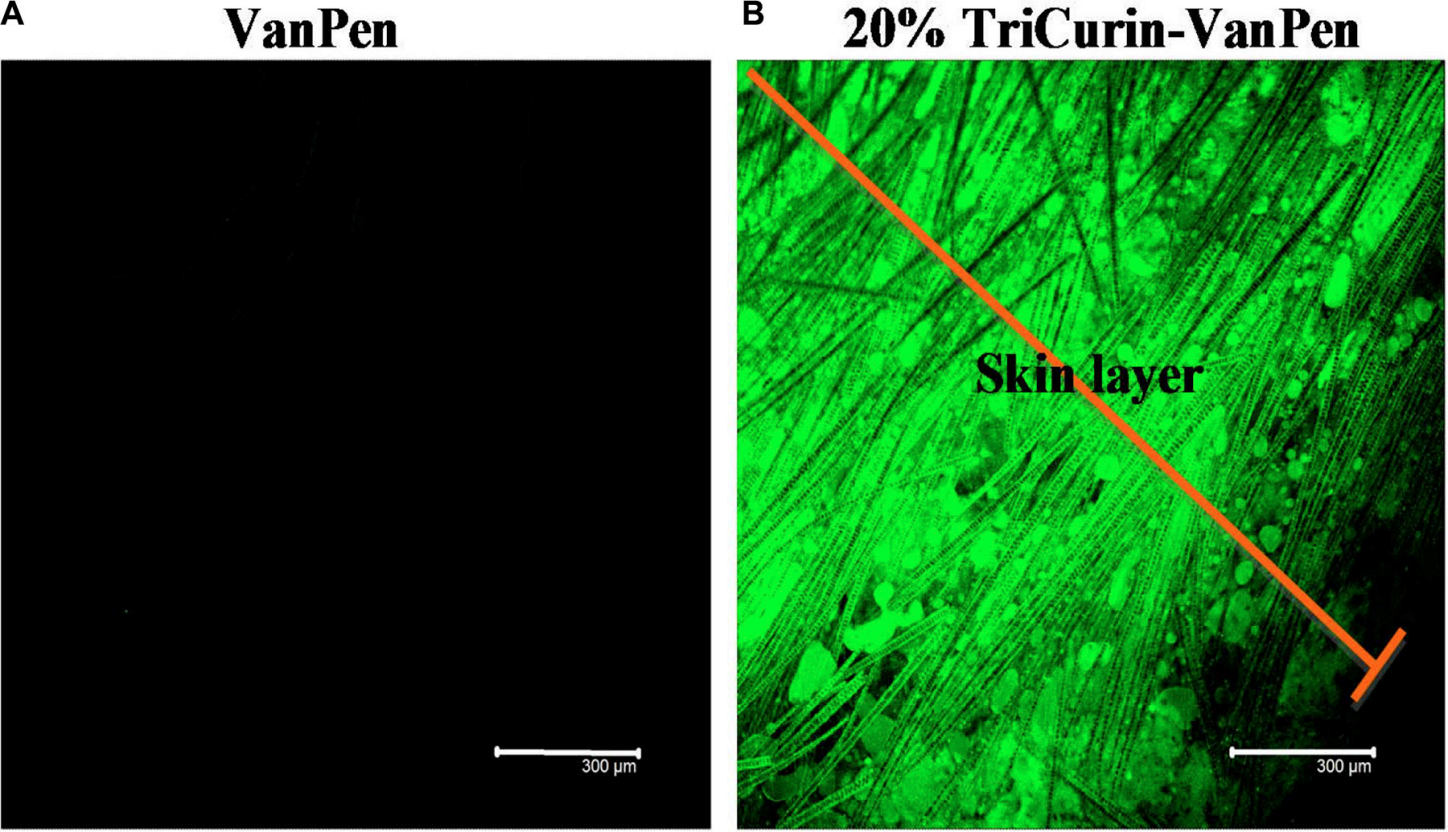
TriCurin-VanPen applied topically allows permeation of curcumin through the skin VanPen or 20% TriCurin-VanPen were applied on shaven skin at the nape of the neck. After 20 h skin sections were examined using confocal microscopy. (**A**) VanPen-treated skin shows no fluorescence. (**B**) TriCurin-VanPen treated skin shows permeation of curcumin through the skin.

To evaluate the therapeutic applicability of TriCurin in the form of a topical or vaginal cream, we applied a uniform mixture of 20% TriCurin in a topical cream base VanPen (TriCurin-VanPen) on the shaven skin of mice. After 20 hours, the mice were terminated by injecting ketamine-xylazine, the skin from the application site removed, sectioned into transverse sections across the thickness of the skin, and the sections examined at 540 nm (emission) using a confocal microscope. As shown in Figure 8, complete permeation of C, which is fluorescent, was observed across the entire thickness of the skin.

We also diluted 1 ml of the 20% TriCurin-VanPen (163 mM+ in TriCurin) 5000-fold in cell culture medium to achieve 32 μM+ TriCurin, from which decreasing concentrations of TriCurin were obtained by serial dilution for the treatment of HeLa cells in culture. Subsequent WST-1 assays faithfully reproduced the IC50 values observed with free TriCurin (Table 1), whereas similarly diluted VanPen had no effect on the HeLa cells. Thus, TriCurin does not lose its antitumor activity in the TriCurin-VanPen formulation.

## DISCUSSION

In the current study, we have improved the efficacy of curcumin (C) as a therapeutic agent against HPV infection and cancer by combining it with two other polyphenols at a unique proportion to yield a synergistic combination, named TriCurin. The crucial factor for this synergism is the proportion of the three polyphenols in TriCurin (C:E:R: 4:1:12.5). While maintaining this proportion, increased efficacy of TriCurin over and above each of the three components and CE or CR was observed at 4 μM+, 8 μM+, and 16 mM+, beyond which individual polyphenols, CE, CR, as well as TriCurin approached a plateau revealing the elimination of almost all HeLa cells in *in vitro* cultures (Figure 1).

HPV E6 inhibits P53 through two mechanisms, one of which entails P53 ubiquitination and degradation *via* E6-P53 association [20]. A second pathway involves E6 binding to histone acetyltransferease (HAT) p300 and its co-activator CBP, thereby inhibiting the ability of p300 to activate the transcriptional activity of both p53 as well as NF-kB [18]. However, NF-kB affords tumor cell protection by inducing Bcl-2 expression, whereas p53 promotes apoptosis by boosting the expression of the pro-apoptotic protein Bax [21]. Curcumin causes inactivation of the transcription factors AP-1 and NF-kB, which have been implicated in the transcription of HPV-associated proteins such as E6 and E7 [22]. This curcumin-evoked inhibition of NF-kB and the consequent suppression of E6 [21], leads to induction of p53, suppression of the NF-kB-Bcl-2 protective pathway, and preferential stimulation of the p53-Bax apoptotic pathway in cancer cells [19]. Corroboratively, we observed a dramatic activation of caspase-3 concomitant with 32-μM+ TriCurin-evoked suppression of E6 in the TC-1 cells (Figure 3). The suppression of E6 and activation of caspase-3 was also observed in the presence of 32-μM C, but the TriCurin effect was several-fold greater than that yielded by C alone (Figure 3). According to the studies discussed above the TriCurin-evoked suppression of E6 should cause decreased degradation of p53. Accordingly, we observed a 13-fold induction of p53 in the 32-μM+ TriCurin-treated TC-1 cells with respect to the vehicle-treated cells (Figure 4). It is known that C-terminal acetylation of p53 by the HAT p300 boosts p53’s transcriptional activity [17, 23]. Correspondingly, 32-μM+ TriCurin caused a 49-fold increase in acetyl-p53 with respect to the vehicle-treated control (when normalized to total p53) (Figure 4J), but since the total p53 expression had also increased by 13-fold, the overall increase in acetyl-p53 was 637-fold with respect to the vehicle-treated control (Figure 4L). Curcumin (32 μM) treatment also caused suppression of E6, activation of caspase-3, and induction in p53 as well as acetyl-p53 with respect to vehicle treatment of TC-1 cells, but the TriCurin-evoked changes were dramatically greater in each case. Based on the reports discussed earlier, our findings indicate that TriCurin or curcumin-evoked suppression of E6 releases p53 from its inhibitory influence thereby allowing active p53 to trigger apoptosis in the TC-1 cells (Supplementary Figure S1).

The reason why TriCurin is strikingly more potent than curcumin is not fully clear. Data presented in Figure 6 and Supplementary Figure S2 indicate that this higher potency of TriCurin could be due to a combination of stabilization as well as increased uptake of C into cells. The current study presents a systematic analysis of combination indices to arrive at this synergistic, highly potent and unique anticancer formulation (TriCurin), but further theoretical and spectroscopic analyses are required to understand how curcumin, resveratrol, and epicatechin gallate at this unique molar ratio interact with one another to yield the dramatically higher anticancer and anti-HPV properties of TriCurin.

In the animal studies, high efficacy of tumor elimination was observed when aliquots of a 1.28 mM+ TriCurin were injected into the tumor to yield an intra-tumor concentration of approximately 64 μM+. A dramatic inhibition of TC-1 tumor growth was observed with TriCurin. Similar application of TriCurin to tumor-naïve mice had no adverse effect on normal tissue. With the objective of using TriCurin for topical application, we tested our cream formulation TriCurin-VanPen, which contained 20% TriCurin solubilized in the widely used cream base VanPen. Application of this TriCurin-VanPen to mouse skin demonstrated complete penetration of curcumin within 20 hours, thus justifying the possibility of its use in topical applications to eliminate warts. Recently, we have also developed a cream base with FDA-approved composition for vaginal application. Our formulation of TriCurin in this cream base will be tested in a human trial for HPV+ cervical dysplasia.

HPV infection leading to cervical cancer has reached almost an epidemic level in many parts of the developing world where appropriate vaccination programs are not affordable for the pre-infection individuals and screening programs are not available for the HPV-infected men and women. Although HPV infection clears spontaneously with time in most younger women [24], some older individuals become persistently HPV+ even in the developed countries. In women, loop electrosurgical excision procedure (LEEP) is commonly used to treat high-grade dysplasia. Though highly effective, LEEP is associated with the risk of cervical incompetence and preterm delivery [25].

In the midst of this serious global health problem due to HPV infection, TriCurin comes as a relatively less expensive but highly effective therapeutic alternative. It potently suppresses HPV E6 expression (Figure 3), efficaciously eliminates HPV+ cells in culture (Figure 1, Table 1), and suppresses HPV E6 and E7+ TC-1 tumors in a dramatic manner (Figure 5). When incubated in the culture medium, curcumin alone rapidly loses its ability to eliminate HeLa cells, but in the TriCurin formulation, curcumin retains its efficacy to eliminate HeLa cells (Figure 6). Based on such properties, the use of TriCurin and its appropriate formulations come as a relatively inexpensive but highly promising therapeutic approach to treat HPV infections and cervical cancer.

## MATERIALS AND METHODS

### Animals

Mice (C57BL6) were handled and used according to an experimental protocol that followed NIH guidelines for animal use and was approved by the Institutional Animal Care Committee (IACUC) of the College of Staten Island (CUNY).

### TC-1 cells

Originally created by retroviral transduction of primary lung epithelial cells from C57BL/6 mice to express the oncogenes HaRas, HPV16 E6 and E7, the TC-1 cell line was a kind gift from Prof. T.-C. Wu (Johns Hopkins Medical Institutions, Baltimore) [16].

### Determination of IC50 for curcumin using WST-1 assay

TriCurin was serially diluted into DMEM plus 1× insulin-transferrin-selenium (ITS) supplement (Invitrogen) and was added to the cells in 50-µl aliquot/well. Subsequent to TriCurin treatment in triplicate wells for 96 hours, the medium was aspirated, the cells rinsed three times with PBS, and then 50 µl of 10% WST-1 (Clontech, Mountain View, CA) in DMEM was added to each well. The plate was incubated at 37°C for 45 min and absorbance monitored at 440 nm using a plate reader. Results obtained were converted to percent control and then analyzed using GraphPad Prism.

### TriCurin

Curcumin (C) (≥ 98% curcuminoid content) (CAS number 458-37-7) (Thermo Fisher Scientific, New Jersey, U.S.A.; prepared by Acros Organics for Thermo Fisher; stored at room temperature under nitrogen), (-)-epicatechin gallate (E), and resveratrol (R) (Thermo Fisher) at the molar proportion C:E:R: 4:1:12.5 was prepared at various doses. A dose that contained C: E: R: (in µM) 32: 8: 100, was named as 32 µM+ TriCurin. First a solution of 1.28 mM+ TriCurin in PBS plus 5% DMSO was prepared by dilution from solutions of C and R in DMSO and a solution of E in PBS. Then, 64 µM+, 32 µM+, 16 µM+, 8 µM+, 4 µM+ TriCurin solutions were prepared through serial dilution of the 1.28 mM+ in serum-free culture medium or PBS. The first two dilutions required addition of small increments (2 µl) of the stock solution into an appropriate volume of medium or PBS with vigorous and continuous vortexing.

### Determination of combination index using the COMPUSYN software package

Dose response analysis for each polyphenol (C, E, and R) and the combinations CE, CR, and CER was performed using the HeLa cells. Fraction affected at each concentration of C (in μM: 4, 8, 16, 32), at each of the corresponding concentrations of E (in μM: 1, 2, 4, 8), R (in μM: 12.5, 25, 50, 100), and at each of the combinations CE, CR, and CER at the increasing combined doses was determined. This series of data (Fractions affected) were next analyzed using the COMPUSYN software package from ComboSyn, Inc. (www.combosyn.com) to determine the combination index at each of the increasing doses of CER (4 μM+, 8 μM+, 16 μM+, 32 μM+) [15]. Using a similar approach, combination indices for equal ratios C:E:R: 8 μM: 8 μM: 8 μM and C:E:R: 32 μM: 32 μM: 32 μM were also determined.

### Western blot analysis

Cells were lysed in RIPA buffer (50 mM Tris-HCl, pH 8.0, 1% NP-40, 0.5% deoxycholate, 0.1% SDS, 1 mM EDTA, 1 mM EGTA 1 mM PMSF, 50 mM NaF, 100 mM Na_4_P_2_O_7_, 10 mM Na β-glycerophosphate, 1 mM Na_3_VO_4_, 1X protease inhibitor cocktail (Roche) and quantified by Lowry protein assay. Equal amounts of protein (20 μg) for all the cells described above were prepared by heating samples in loading buffer composed of 95% Laemmli sample buffer and 5% 2-mercaptoethanol (Sigma, St. Louis, MO) in boiling water for 5 min. The proteins were separated using 10% SDS polyacrylamide gel electrophoresis at 100 V for 1.5 h. The electrophoretic transfer of proteins onto a 0.45-μm nitrocellulose membrane was performed at 200 mA constant current for 2 h on ice. Primary antibody solutions were prepared in Tris-buffered saline containing Tween (T-TBS; 20 mM Tris buffer, pH 7.4, 150 mM sodium chloride, 0.05 % Tween-20) and 1% non-fat dried milk and incubated overnight. The antibody against p53 (sc-6243) was obtained from Santa Cruz Biotechnology (Santa Cruz, CA) and a mouse monoclonal ß-actin antibody (SKU A2228) was procured from Sigma (St. Louis, MO). All the primary antibodies were used at a dilution 1:1000 except for the ß-actin antibody, which was used at a dilution of 1:5000. The secondary antibodies, anti-rabbit or anti-mouse were used at dilutions 1:40,000 and 1:5000 respectively. The Western blot signals were visualized using enhanced chemiluminescence (Super Signal West Pico, Pierce, Rockford, IL) and digitally imaged with an Alpha Innotech imager (San Leandro, CA). The protein bands were quantified using ImageJ and the figures were arranged using Adobe Photoshop.

### Immunocytochemistry

HeLa and TC-1 cells (60,000 cells per well) were plated in triplicate wells on poly-L-lysine-coated coverslips in 24-well plates and treated with drugs after the wells reached 80% confluence. All drug treatments were performed in serum-free DMEM containing 1% supplement (ITS) (insulin, transferrin, selenium; Gibco BRL, Grand Island, NY). After treatment with 32 µM+ of TriCurin or C for the indicated times, cells were fixed in 4% paraformaldehyde for 45 minutes at room temperature, rinsed with phosphate buffered saline (PBS) three times (15 min each), and blocked with 10% normal goat serum, 0.1 % Triton X-100 in PBS for 2 h. The cells were then incubated overnight with anti-E6 antibody (sc-460, Santa Cruz Biotechnology, Dallas, TX) (1:75) in 2% normal goat serum, 0.1 % Triton X-100 in PBS. In mechanistic studies, the TC-1 cells were treated with anti-E6, anti-p53 (sc-6243, Santa Cruz Biotechnology, Dallas, TX) (1:100), anti-acetyl-^379^K-p53 (GTX88013, GeneTex, Inc., Irvine, CA) (1:300), and anti-active caspase-3 (CST#9661, Cell Signaling Technology, Danvers, MA) (1:200) antibodies in a similar manner. The acetylated ^379^K position on mouse p53 is homologous to the ^382^K acetylation site on the human protein [26, 27]. Subsequent to primary antibody treatment, the cells were washed three times with PBS and then incubated with the respective secondary antibodies (Alexa Fluor^®^ 488 goat anti–mouse IgG, Alexa Fluor^®^ 488 goat anti–rabbit IgG and Alexa Fluor^®^ 568 goat anti–rabbit IgG antibodies) (Life Technologies Corp., Carlsbad, CA) in 2% normal goat serum, 0.1 % Triton X-100 in PBS (1:1000) for 3 h and then washed three times with PBS (15 min each) followed by incubation with HOECHST33342 (HOECHST) (10 µg/ml) in PBS (10 µM) for 30 min and three washes with PBS. The coverslips containing stained cells were mounted on slides and cell images were acquired using a Zeiss Axio Observer Z1 microscope and an AxioVision 4.6.3-AP1 camera at emission wavelengths of 460 nm (blue) 540 nm (green) and 580 (red). Images of two different, randomly chosen fields were acquired from each well for quantification. ImageJ (NIH, Bethesda, MD) was used to measure the E6, p53, acetyl-^379^K-p53, active caspase-3 and HOECHST fluorescence intensities. The fluorescence intensities of all the above-mentioned antibodies were normalized to HOECHST intensity (blue). Since p53 displayed both induction as well as acetylation-mediated activation, the HOECHST-normalized staining intensities were expressed as acetyl-^379^K-p53/p53, p53/HOECHST and acetyl-^379^K-p53/HOECHST.

### In vivo studies to demonstrate anti-tumor activity of TriCurin

TC-1 cells generated by engineering mouse lung epithelial cells to ras and HPV16/18 E6 and E7 have been used earlier to generate preclinical mouse models of HPV+ cancer [16]. In our studies 50,000 TC-1 cells were implanted subcutaneously at the nape of the neck of two to three month-old C57BL6 mice. When the tumors assumed the approximate length of 0.5 cm, each tumor was marked into four quadrants and 2.5 µl of the 1.28 mM+ TriCurin solution was infused into each of the four quadrants every 72 hours (final estimated concentration in the tumor: 64 μM+). Intralesional 1.28 mM+ TriCurin or vehicle (PBS plus 5% DMSO) treatment was conducted on days 1 (i.e. at approximate length of 0.5-cm), 4, and 7. Tumor dimensions were measured using a caliper before each treatment and in the end, 72 hours after the third TriCurin treatment (tumor dimension = length × width × height × 0.5). Subsequently, the mice were sacrificed, tumors extricated, and the final tumor volumes were measured again by displacement of water.

### Toxicity tests for TriCurin

Three 3-month-old female mice in each of two groups were administered (subcutaneously, at the nape of the neck) PBS, or 10 μl 1.28 mM+ TriCurin (every 72 hours) for two weeks. Subsequently, the injection area of each mouse was dissected into tissue sections, which were stained with hematoxylin and eosin (H&E) for histopathologic examination by a pathologist (L.M.O).

### Topical TriCurin-VanPen cream

Curcumin 1.5 g, Epicatechin Gallate 0.5 g, Resveratrol 3.0 g and 20.0 g VanPen base cream (Professional Compounding Centers of America, Inc., PCCA, Houston, TX) were mixed thoroughly as follows: 10 g of VanPen was placed in the mixing chamber of a pharmaceutical-grade mixer (Electronic Mortar and Pestle Ungulator 2100 by GAKO International, München, Germany), C, E, R, powders were added to it, then 10 g of VanPen was added on top of the mixture, and the ingredients were mixed thoroughly with a motor-driven rod with blades until a uniform, yellow emulsion was obtained. This yielded approximately 25 ml of the 20% TriCurin-VanPen cream (163 mM+ in TriCurin).

### Statistical analysis

Statistical analyses were performed using Microsoft Excel^®^ 2007 (Microsoft Corporation, Redmond, WA) and GraphPad Prism^®^ 5 (GraphPad Software, Inc., La Jolla, CA). Means and standard deviations were calculated for each treatment and converted to percent carrier-treated. Half maximal inhibitory concentration (IC50) values were obtained after performing regression analyses. Significance was assessed using two-tailed *t-test* with unequal variance for comparison between two groups and one-way ANOVA for comparison among three or more data sets using Tukey for post-hoc analysis. In both analyses, *p* < 0.05 was considered as significant.

## SUPPLEMENTARY MATERIALS FIGURES


