# Supplementary material for: Unique synergistic formulation of curcumin, epicatechin gallate and resveratrol, tricurin, suppresses HPV E6, eliminates HPV+ cancer cells, and inhibits tumor progression
Source: Oncotarget. 2017 Mar 29;8(37):60904–16. doi: 10.18632/oncotarget.16648 (PMC5617393; doi:10.18632/oncotarget.16648)
Supplement: Supplementary file 1 [file oncotarget-08-60904-s001.pdf]

Unique synergistic formulation of curcumin, epicatechin gallate and resveratrol, tricurin, suppresses HPV E6, eliminates HPV+ cancer cells, and inhibits tumor progression

SUPPLEMENTARY MATERIALS

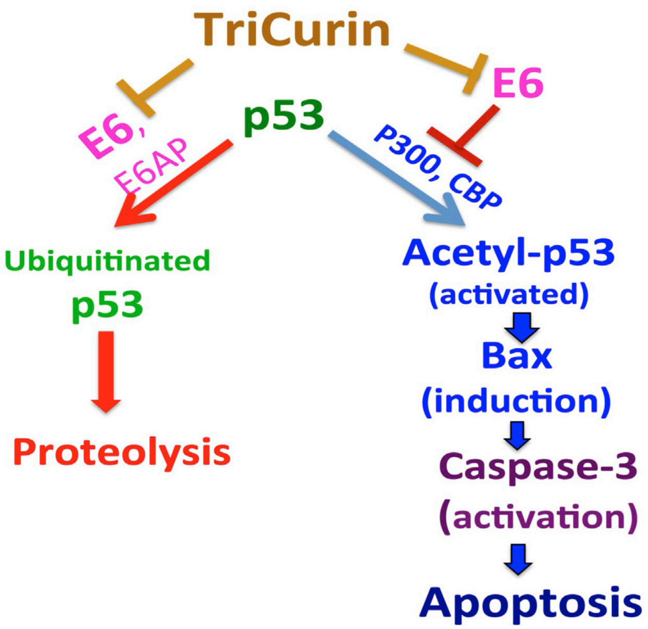

Supplementary Figure S1: Mechanism of TriCurin mediated regulation of p53 and elimination of TC-1 cells. HPV E6 regulates p53 by (i) binding, sequestration and ubiquitination of p53 followed by its proteolysis, and (ii) inhibiting the binding of p300 (HAT) and CBP to p53, which causes acetylation and activation of p53. TriCurin or curcumin (at a lesser potency) causes suppression of E6 expression, thereby releasing p53 from the inhibitory influences of E6 and causing both induction as well as increased activation of p53.

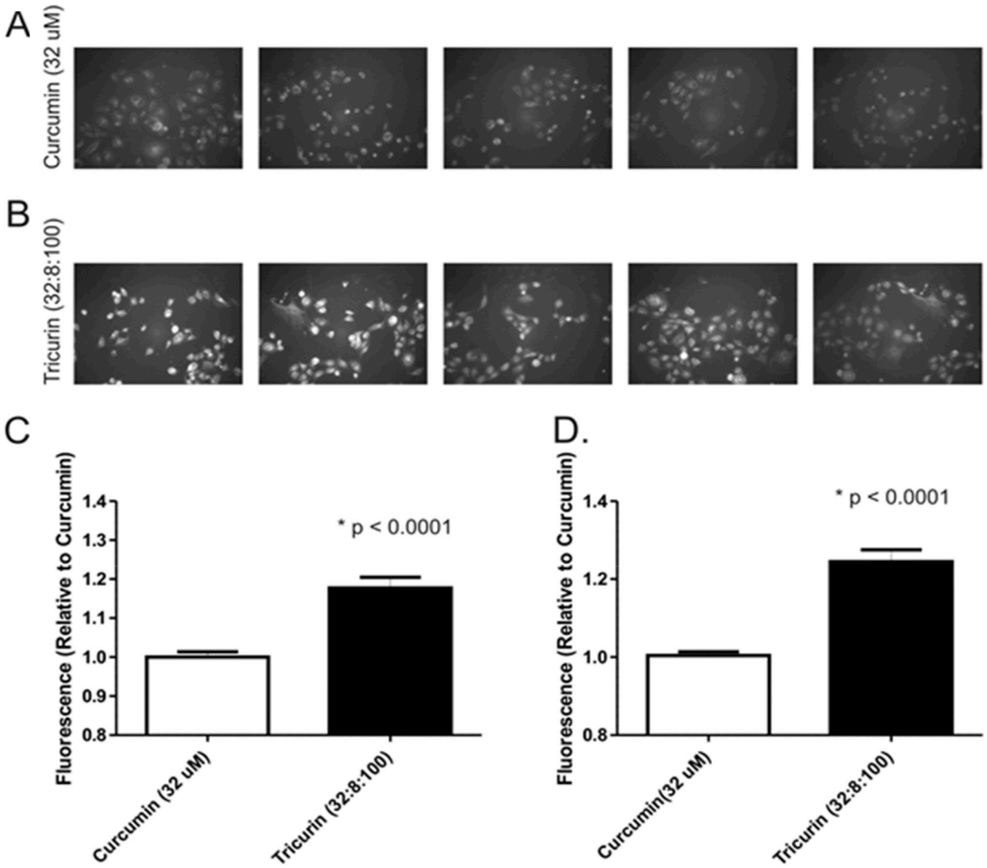

Supplementary Figure S2: TriCurin significantly enhances cellular intake of curcumin. Cells were incubated for four hours with either curcumin (32 µM) or TriCurin (32 mM+). Following incubation, cellular uptake of curcumin was captured using fluorescence imaging (**A** and **B**). Cells incubated with TriCurin displayed significantly higher fluorescence (17.8%) than cells incubated with curcumin alone ($p < 0.0001$; *t*-test) (**C**). When curcumin and Tricurin were incubated in solution overnight at 37°C and then added to cells, the cellular uptake of curcumin from the TriCurin solution was 25% higher than that from the incubated curcumin solution ($p < 0.0001$; (**D**). This indicates that Tricurin enhances cellular uptake of curcumin and may protect curcumin from oxidation while in solution. No fluorescence was noted with (–)-epicatechin gallate or resveratrol. The fluorescence of curcumin was imaged using excitation at 450 nm. Data shown here are representative of three independent experiments.
